# A Systematic Review and Meta-Analysis on the Role of Nutraceuticals in the Management of Neuropathic Pain in In Vivo Studies

**DOI:** 10.3390/antiox11122361

**Published:** 2022-11-28

**Authors:** Sara Ilari, Stefania Proietti, Patrizia Russo, Valentina Malafoglia, Micaela Gliozzi, Jessica Maiuolo, Francesca Oppedisano, Ernesto Palma, Carlo Tomino, Massimo Fini, William Raffaeli, Vincenzo Mollace, Stefano Bonassi, Carolina Muscoli

**Affiliations:** 1 Department of Health Science, Institute of Research for Food Safety & Health (IRC-FSH), University “Magna Graecia” of Catanzaro, 88100 Catanzaro, Italy; 2 Clinical and Molecular Epidemiology, IRCCS San Raffaele Roma, 00166 Rome, Italy; 3 Department of Human Sciences and Quality of Life Promotion, San Raffaele University, 00166 Rome, Italy; 4 ISAL Foundation Institute for Research on Pain, Torre Pedrera, 47922 Rimini, Italy; 5 Scientific Direction, IRCCS San Raffaele Roma, 00166 Rome, Italy

**Keywords:** neuropathic pain, oxidative stress, antioxidants, new therapeutic targets, systematic review, meta-analysis

## Abstract

The control of neuropathic pain is a leading challenge in modern medicine. Traditional medicine has, for a long time, used natural compounds such as nutraceuticals for this purpose, and extensive evidence has supported their role in controlling oxidative stress and persistent pain-related inflammation. Nutraceuticals are natural products belonging to the food sector whose consumption could be related to physiological benefits. Indeed, they are used to improve health, prevent chronic diseases, and delay the aging process. Here, we report a systematic review and meta-analysis to provide a more comprehensive report on the use of nutraceuticals in neuropathic pain, including evaluating confounding factors. A search of the literature has been conducted on principal databases (PubMed, MEDLINE, EMBASE, and Web of Science) following the PRISMA statement, and we retrieved 484 articles, 12 of which were selected for the meta-analysis. The results showed that administration of natural drugs in animals with neuropathic pain led to a significant reduction in thermal hyperalgesia, measured in both the injured paw (SMD: 1.79; 95% CI: 1.41 to 2.17; *p* < 0.0001) and in the two paws (SMD: −1.74; 95% CI: −3.36 to −0.11; *p* = 0.036), as well as a reduction in mechanical allodynia and hyperalgesia (SMD: 1.95, 95% CI: 1.08 to 2.82; *p* < 0.001) when compared to controls. The results of the review indicate that nutraceutical compounds could be clinically relevant for managing persistent neuropathic pain.

## 1. Introduction

### 1.1. Neuropathic Pain

Neuropathic pain is a serious health problem affecting millions of people in the world, representing a debilitating condition which is often overwhelming for patients.

According to the International Association for the Study of Pain (IASP), neuropathic pain is the “pain caused by injury or disease of the somatosensory system” [1]. The main characteristics of this condition are allodynia (pain caused by painless stimuli) and/or hyperalgesia (increased response to noxious stimuli) in addition to continuous development (i.e., independent of the stimulus) [2,3]. The mechanisms of induction and maintenance of chronic neuropathic pain are still poorly understood [4,5]. However, it is well known that in tissue injury, hyperalgesia and allodynia lead to a persistent state of peripheral sensitization that subsequently initiates spinal sensitization. 

Various diseases may induce neuropathic pain following nerve lesions, i.e., autoimmune diseases (multiple sclerosis), metabolic diseases (diabetic neuropathy), infections (postherpetic neuralgia), vascular disease (stroke), trauma, and cancer [6]. 

Although several pharmacological and non-pharmacological approaches have been proposed [7], current treatments are not entirely adequate to relieve neuropathic pain, and novel options are, therefore, necessary. Treatment options for neuropathic pain include first-line drugs, such as antidepressants (tricyclic agents, TCA) [8,9] and anticonvulsants (gabapentin and pregabalin). These exert their mechanism of action through the potential calcium channel-blocking effects [10], without binding to GABA receptors or interacting with GABA uptake transporters [11,12]. Opioids are the most effective treatments for severe chronic pain. Their prolonged use is limited by the development of dependence, analgesic tolerance, and, thus, oxidative stress. Nevertheless, they are second-line drugs to treat neuropathic pain [13], since their effectiveness requires increasing doses to achieve similar pain relief. This limits their use for chronic management of neuropathic pain [14]. Table 1 describes the treatment options for neuropathic pain.

In addition, non-pharmacological treatments for chronic pain are also increasingly used, including behavioral, cognitive, integrative, and physical therapies [7]. Other techniques used to treat chronic and neuropathic pain include the classic method of neural blockade therapy. For instance, neural blockade of the scalp can be used as an anesthetic for intra- or extracranial procedures or as general anesthesia [15]. In Europe, neural stimulation is heavily used, and includes spinal cord stimulation (SCS), peripheral nerve stimulation (PNS), transcutaneous electrical nerve stimulation (TENS), and motor cortex stimulation (MCS) [15].

The interest in drugs of natural origin is dramatically increasing, due to the fewer side effects, lower costs for national health systems, and patients’ trust in natural products [16,17]. In addition, evidence shows that natural antioxidants act against oxidative stress generated immediately after the insurgence of neuropathic disease [18].

### 1.2. Oxidative Stress in Neuropathic Pain

The pathways involved in developing neuropathic pain are complex, and numerous molecular and cellular mechanisms are involved. Oxidative species formation is critical for the onset of neuropathic pain and neurodegenerative diseases, including diabetes, cancer, premature aging, osteoarthritis, atherosclerosis, and Crohn’s disease [19]. 

Increased extracellular glutamate levels following painful stimuli lead to the activation of numerous intracellular pathways, including free radicals’ formation (oxygen (ROS) and nitrogen (RNS) reactive species). In particular, free radicals increase during stress, resulting in oxidative/nitroxidative stress. This condition activates cyclooxygenase (COX) enzymes and enhances the production of prostaglandins through the activation of transcription factors (i.e., activator protein 1 (AP1), nuclear factor kappa (NF-kB)), and mitogen-activated protein kinases (MAPK) [20,21]. Inflammatory cell activation, together with cytochrome P450 metabolism, is a potential source of endogenous ROS production. Superoxide anion (SO) is considered the “primary” ROS, and mitochondrial SO production increases with the presence of diseases. Levels of SO are kept under control by the manganese superoxide dismutase (MnSOD) isoform in the mitochondrion, and by the copper, Zinc-SOD (Cu, ZnSOD), in the cytoplasm [22,23]. Furthermore, SO and peroxynitrite (PN), the main agents responsible for peripheral and central sensitization [24,25,26,27], stimulate the production of pro-inflammatory cytokines (TNF-α, IL-1β, IL-6), prostanoids, nitric oxide, excitatory amino acids (i.e., glutamate). In addition, they activate spinal cord glial cells, damage endothelial cells, recruit neutrophils, cause single-stranded DNA damage, activate poly-ADP-ribose-polymerase (PARP), and, completing a vicious cycle, contribute to the formation of PN and lipid peroxidation products. 

**Figure 1:**
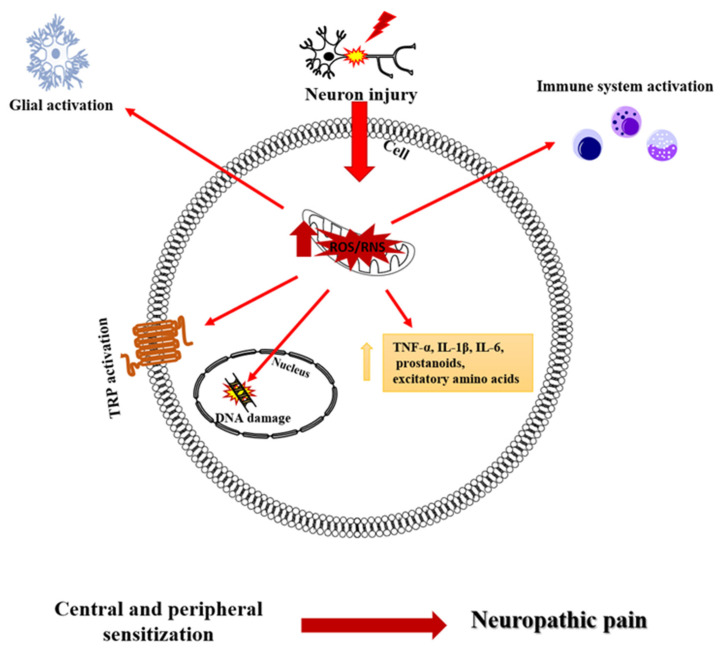
Free radical action following nerve injury.

The direct role of SO and PN in the onset of neuropathic pain has been demonstrated by the development of thermal hyperalgesia following intraplantar administration of SO and PN. Furthermore, this hyperalgesic state was accompanied by the development of chronic inflammation following the spinal activation of the N-methyl-D-aspartate receptor (NMDAR) [24] (Figure 1).

An important ROS/RNS target is polyunsaturated fatty acids, which are extremely sensitive to oxidation. The final products of this reaction, which is referred to as lipid peroxidation, are malondialdehyde (MDA) and 4-hydroxynonenal (4-HNE) [3,21]. Oxidative stress occurs when ROS/RNS production overwhelms a cell’s antioxidant capability, leading to cellular macromolecule damage-causing alteration of the mitochondrial pathway. This event is, in turn, responsible for the post-translational modification of key proteins, such as mitochondrial manganese superoxide dismutase (MnSOD), glutamate transporter (GLT-1), glutamate receptor N-methyl-D-aspartate (NMDA), and glutamine synthase (GS) [28].

The excess of free radicals is generally inactivated by the endogenous or exogenous antioxidant activity of molecules that inhibit the oxidation by directly removing free radicals, thus providing maximal protection for biological sites.

### 1.3. Natural and Synthetic Antioxidants

Antioxidants are classified according to their chemical nature into enzymatic and non-enzymatic antioxidants. Enzymatic antioxidants remove free radicals, converting dangerous oxidative products into hydrogen peroxide (H2O2) and water. The most efficient endogenous enzymatic antioxidants include superoxide dismutase (SODs), glutathione peroxidase, and catalase (CAT). SODs, a class of oxidoreductase enzymes that contain Cu/Zn or Mn at the active site, catalyze the dismutation of superoxide to H2O2 and are the first line of defense against free radicals. 

The enzyme is present in all aerobic organisms and subcellular compartments susceptible to oxidative stress [29]. In mammals, three isoforms of SOD have been discovered to be localized within the cell: SOD1, or Cu/Zn SOD, is found mainly in the cytoplasm and nuclear compartments [22,30]; SOD2, or MnSOD, is in the mitochondria; SOD3, or EC-SOD, is in the extracellular compartments [31]. Glutathione peroxidase can reduce lipid hydroperoxides to their corresponding alcohols and reduce free hydrogen peroxide to water [32]. Catalase, in the peroxisome, converts hydrogen peroxide into water and molecular oxygen. It also binds to NADPH in order to prevent oxidative enzyme inactivation by H2O2 when reduced to water [33,34]. 

Non-enzymatic exogenous antioxidants can be classified into natural antioxidants and synthetic antioxidants. Synthetic antioxidants are substances created from chemical processes, and can be used to remove free radicals, as reported by our recent studies [2,35]. Among them, Mn (III)tetrakis (4-benzoic acid) porphyrin (MnTBAP) is a potent inhibitor of lipid peroxidation [36,37]. Moreover, it protects against some effects associated with endotoxic and hemorrhagic shock, as well as acute and chronic pain [21,38]. Its efficacy is likely due to its SO and PN scavenging activity. Another extensively studied synthetic antioxidant is the metalloporphyrin iron tetrakis (N-methyl-4’-pyridyl) porphyrin (FeTMPyP). Intrathecal injection of PN decomposition catalyst FeTMPyP prevents nitration and inhibits NMDA-mediated thermal hyperalgesia [2,28]. On the other hand, natural antioxidants are obtained from natural sources, and are generally used in the food, cosmetics, and pharmaceutical industries; they include low-molecular-weight compounds, such as vitamins, plant-derived polyphenols, glutathione, and carotenoids. Vitamins (especially C and E) have good antioxidant properties, although recommended daily levels of vitamins are insufficient to prevent oxidative damage to cells [39]. Particularly, vitamin C (ascorbic acid) acts directly by eliminating superoxide, hydroxyl radicals, and singlet oxygen, or by reducing H2O2 to water through the ascorbate peroxidase. It can also regenerate vitamin E (tocopherol) from the α-tocopheroxyl radical, protecting the membrane [40]. α-tocopherol is the most active form of vitamin E, and may directly repair oxidative radicals, preventing the chain propagation step during lipid autoxidation. It reacts with peroxyl lipid radicals and alkyl radicals derived from polyunsaturated fatty acids (PUFA) oxidation [41,42]. Glutathione (GSH) is the major soluble antioxidant, and the GSH/GSSG ratio is a principal target of oxidative stress. GSH can eliminate cytotoxic H2O2 and reacts non-enzymatically with other ROSs: single oxygen, superoxide radical, and hydroxyl radical. In addition, the GSH antioxidant role is to regenerate another powerful water-soluble antioxidant, i.e., ascorbic acid, through the ascorbate–glutathione cycle [43,44]. Finally, carotenoids (i.e., Lycopene and β-carotene) are fat-soluble phytonutrients known to eliminate peroxyl radicals, and they play an essential role in protecting lipoproteins and cell membranes against ROSs [45].

Nutraceuticals are natural products belonging to the food sector, particularly used as food supplements. The term “nutraceutical” is derived from “nutrition” and “pharmaceutics”, because they are very often formulated as concentrated or purified products, administered as pills, tablets, or other pharmaceutical forms, and because they may exert some health benefits [46].

In recent years, the use of nutraceuticals rich polyphenols has increased significantly for pain treatment, thanks to their lower side effects compared to pharmaceuticals. Polyphenols are micronutrients with a wide range of beneficial effects on humans, involved in the prevention of inflammatory and neuropathic pain, neurodegenerative diseases, cardiovascular diseases [47], diabetes, and osteoporosis [48,49] (Table 2).

**Figure 2:**
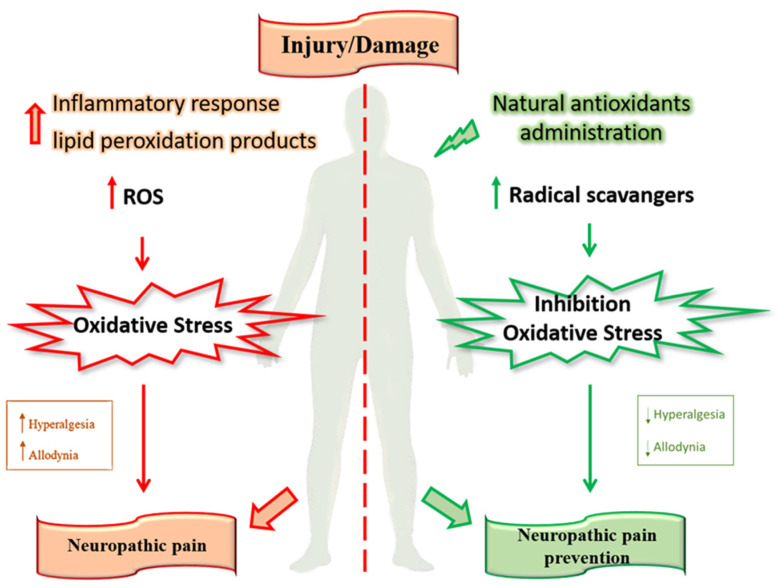
Role of natural antioxidants in pain prevention.

Polyphenols are involved in improving endothelium dysfunction, metabolic function, and the body’s antioxidant defenses. Furthermore, the number of phenolic rings and structural elements that bind these rings together classifies polyphenols [50]. Among these, the most important compounds are flavonoids and stilbenes in fruits and vegetables [51,52]. These are involved in various biological functions, including the modulation of a wide range of enzymes and cell receptors and the protection from DNA damage [53]. Natural products, mainly medicinal plants, are one of the oldest forms of medical practice to cure and prevent diseases. More recently, natural antioxidants have contributed to developing new drugs for managing chronic pain, including neuropathic pain [45,47,52,54,55] (Figure 2).

We report a systematic review and meta-analysis of the existing literature on animal studies, in which we examine the mechanisms of action of different nutraceuticals in treating neuropathic pain and assess the influence of confounding factors. This study evaluated several animal models in order to include all the possible known therapies, considering the limited efficacy of current therapies for chronic pain. 

The present meta-analysis aims to evaluate the effect of natural drugs on neuropathic pain and to investigate whether the animal model of pain, the type of animal, the route of administration, or different pain measurements could influence the results.

## 2. Materials and Methods

### 2.1. Database Sources

The systematic review includes published data from experimental studies regarding the mechanism of action of different nutraceuticals (both in terms of dose and type) on neuropathic pain. The keywords used for the research of the articles were: “natural antioxidants and neuropathic pain”, “nutraceuticals and neuropathic pain”, “polyphenols and neuropathic pain”, “natural drugs and neuropathic pain”, “natural products and neuropathic pain”, and “phytotherapy and neuropathic pain”.

The studies included in the review were retrieved from the PubMed, MEDLINE, EMBASE, and Web of Science databases, in accordance with the PRISMA (Preferred Reporting Items for Systematic Reviews and Meta-Analyses) statement and following the PICOs framework (Population Intervention Comparison Outcome Population) [56]. All papers written in English and published in the period from 2012 to 2022 were evaluated. The reference list of all retrieved articles was also reviewed in order to identify other eligible studies that were not indexed by the aforementioned databases. 

**Figure 3:**
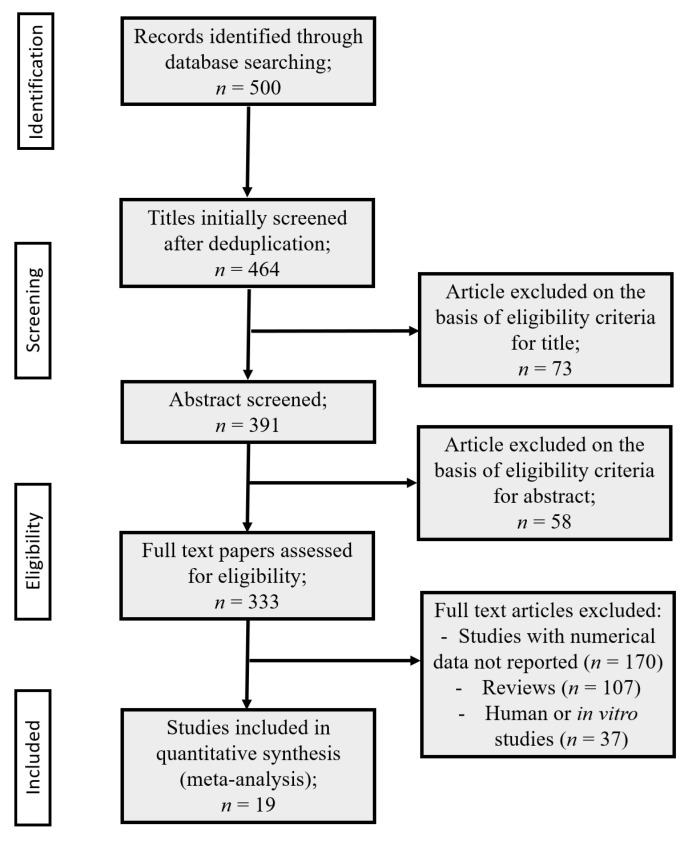
PRISMA flow chart.

As reported in the flow-chart (Figure 3) there were studies matching the inclusion criteria with regard to animals, treatment, and study design, which were not included in the analysis, as numerical data were not available, but only their graphical representations were. For these reasons, we contacted 170 primary or corresponding authors of these studies by e-mail twice. No additional papers were retrieved from this activity. However, to evaluate whether the exclusion of these studies introduced a selection bias, we performed a sensitivity analysis on a random sample of 10 studies [57,58,59,60,61,62,63,64,65], wherein the numerical values of means, medians, SEM, and SD were extrapolated from the graph. The point estimate of the effect (SMD = 1.89) was very close to the results of the meta-analysis described in the paper, though it was not possible to evaluate the variability of the single studies, since the extrapolation of SEM/SD from the graphs was not always possible/reliable. 

### 2.2. Eligibility Criteria

− Studies on animals (rats or mice) with neuropathic pain;
− Study using any natural drug treatments for neuropathic pain.
− Studies were excluded from the analysis for the following reasons:
− use of non-natural drugs or synthetic substances;
− non-pharmacological interventions for pain;
− cellular studies;
− human studies;
− studies in animals other than rats and mice.

### 2.3. Study Outcomes

We performed two separated analyses in order to consider the heterogeneity of outcomes. In the first meta-analysis, the primary outcome was the change in thermal hyperalgesia through paw withdrawal latency (sec), comparing different experimental models of neuropathic pain between animals that received pain only (ctrl group), and those that were also treated with natural drugs (experimental group). 

Specifically, thermal hyperalgesia was examined, considering both the paw withdrawal latency measured in the injured paw and that in both paws (through the difference between the injured and the healthy paw, or through the mean between the two paws).

The outcome of the second meta-analysis was the variation of mechanical allodynia and hyperalgesia through paw withdrawal threshold (gr) in the injured paw, comparing different experimental models of neuropathic pain between animals that received pain only (control group) and those that were also treated with natural drugs (experimental group).

### 2.4. Statistical Analysis

Continuous outcomes, measured on the same scale, are expressed as the mean value and standard deviation, for descriptive purposes. Standardized mean differences (SMD) and the corresponding 95% confidence interval (95% CI) were used to estimate pooled results. The I-square (I2) and Q chi-square tests were performed—according to the Cochrane review guidelines—to evaluate the presence of heterogeneity on the meta-analysis results (when there was a significant heterogeneity with I2 > 50%, random effect modeling was used). Effect sizes are represented by the standardized mean difference, and are presented together with their 95% confidence intervals. SMDs were interpreted as small (0.2–0.5), moderate (0.5–0.8), or large (>0.8), based on [66]. In order to explore possible contributors to the study variance, we investigated the effects of biological and clinical potential confounders on the SMD by univariate meta-regression analysis, reporting a Q chi-square of the moderators’ test (QM). The presence of publication bias was tested using funnel plots adjusted through the “trim and fill” method, as well as Egger’s test. Statistical assessment was two-tailed, and was considered statistically significant at *p* < 0.05. All analyses were carried out using R version 3.6.2, with the packages metafor and meta (metafor; Statistical program).

## 3. Results

### 3.1. Data Collection

The systematic review identified 500 records from the literature search. Thirty-six papers were excluded as duplicates, and seventy-three were excluded due to the contents not fitting the aims of the review. Three hundred ninety-one articles were assessed for eligibility and, after abstract and full text screening, three hundred fourteen had been excluded, as they were reviews, in vitro studies, congress communication, or their dates were incomplete. Finally, 19 papers describing in vivo experiments were included in quantitative analysis (meta-analysis) and split into two meta-analyses according to the behavioral pain assessment method. The process of the literature search and the papers’ screening is illustrated in Figure 3 (PRISMA flow chart [67]).

### 3.2. Systematic Review on the Effect of Nutraceuticals in Neuropathic Pain

Table 3 summarizes the main characteristics of the 19 papers on the use of nutraceuticals in controlling neuropathic pain. Data were based on the effect of nutraceuticals as therapeutic agents in experimental models of neuropathic pain. 

Briefly, bergamot is a natural antioxidant that differs from other citrus fruits due to its composition and its high content of flavonoids. Bergamot from different chemical extracts has been used as an antioxidant to prevent chronic diseases such as neuropathic pain [25,68,69,70]. It also reduces oxidative stress, restoring mitochondrial homeostasis. In particular, bergamot polyphenolic fraction (BPF) restores the biological functions of the mitochondrial proteins SIRT3 (member of the class III histone deacetylase family, HDAC) and MnSOD. Both enzymes are inactivated in rat models of CCI, highlighting the beneficial effect of bergamot during oxidative stress-induced pain [3].

Furthermore, preclinical studies indicated that bergamot essential oil (BEO) could regulate pain perception in different inflammatory, nociceptive, and neuropathic pain models by modulating endogenous antioxidant systems. The administration of BEO, in combination with a low dose of morphine, showed an activation of the peripheral opioid system and induced antiallodynic effects [68]. Komatsu et al., 2018 [69] showed BEO antioxidant effect in a partial sciatic nerve ligation (PSNL) model of neuropathic pain. The intra-plantar injection of bergamot in PSNL mice resulted in the attenuation of allodynia and the inhibition of ERK activation in the spinal cords of mice, probably triggered by the activation of peripheral μ-opioid receptors [69].

Bergamot (*Citrus bergamia*) and N-palmitoyl-D-glucosamine (PGA) are also effective during chemotherapy-induced neuropathic pain. Specifically, in the rat model of chemotherapy-induced neuropathic pain, intraperitoneal injection of BPF protects from thermal hyperalgesia and mechanical allodynia. Hence, the development of neuropathic pain occurs via inhibition of the nitration of essential proteins involved in oxidative stress [3,71].

PGA, a natural molecule produced by *Rhizobium leguminosarum* bacteria and related to the lipid A component of LPS, known for its immunoregulatory and anti-inflammatory properties, has also been observed to prevent damage induced by LPS and peripheral nerve damage by reducing neuropathic pain [72].

*Lepidium meyenii Walp.*, commonly called Maca, belongs to the Brassicaceae family, and is able to restore the pain threshold alterations evoked by oxaliplatin and paclitaxel. For this reason, the compound could be considered a therapeutic opportunity to relieve neuropathic pain [73].

Chlorogenic Acid (5-caffeoylquinic acid, CGA) is a naturally occurring phenolic compound that has antioxidant, anti-inflammatory, and analgesic activity. Indeed, it has been observed that this compound, through the modulation of hyperglycemia and oxidative stress, has the potential to alleviate diabetic neuropathic pain [74]. 

Cannabinoids and endocannabinoid systems are also emerging promising agents to manage the neuroimmune effects of nociception and thermal hyperalgesia [75,76,77]. In particular, Δ9-tetrahydrocannabinol (THC), cannabidiol (CBD), and their combination are being considered as therapeutic alternatives for treatment of NP [75]. Linher-Melville et al., in 2020, studied CBD, THC, and their association. The THC: CBD combination has been observed to provide a beneficial effect in male rats after neuropathic pain induction [75]. Cannabidiol (CBD) is an active phytocannabinoid of *Cannabis sativa* L. and exerts anti-inflammatory and antioxidant properties [78]. Repeated treatment with cannabidiol (CBD) oil positively affected mice’s behavioral dysfunctions associated with traumatic brain injury (TBI). It may be adequate to modulate the immune response following peripheral nerve constriction [77]. In particular, it was observed to be responsible for immunosuppression, starting two months after the nerve injury, at the level of recruitment/proliferation of T lymphocytes in thyme [75].

In addition, endocannabinoid-related N-acylethanolamines, such as palmitoylethanolmide (PEA) and oleoylethanolamide (OEA), have been shown to participate in pain mechanisms [79]. Indeed, Guida et al., in 2015, showed that daily treatment with PEA or OEA reduced pain symptoms in a model of spared nerve injury (SNI) in mice [80], and PEA treatment also reverted the depression and the impaired cognitive function associated with neuropathy [80]. 

Epigallocatechin-3-gallate (EGCG), a polyphenolic compound found in green tea, appears to have numerous beneficial effects due to its antioxidant activity [81]. Some studies have shown a protective effect of EGCG against neurodegenerative diseases, spinal cord injuries, and ischemia. Few studies have been conducted on the antioxidant effect of EGCG in neuropathic pain. Renno et al., in 2012, demonstrated that EGCG improves functional behavioral recovery, protects muscle fibers from cell death by activating anti-apoptotic pathways, and improves morphological recovery in skeletal muscle tissues after nerve injury in rats [81]. Subsequent studies by Xifrò in 2015 [82] and Bosch-Mola in 2017 [83] confirmed the role of EGCG in reducing thermal hyperalgesia through down-regulation of CX3CL1 protein expression. EGCG also inhibited the mechanical allodynia via decreased pro-inflammatory cytokine synthesis and reduced NF-kB activity [82,83].

*Annona muricata Linn.* (Annonaceae), also known as Soursop, is used in traditional African medicine for the treatment of neuralgia, rheumatism, and arthritic pain, and it has also been observed to possess analgesic and anti-inflammatory activities in various animal models of pain [84].

Plant products for treating pain conditions, including diabetic neuropathy, have been widely used in recent years. For example, rosmarinic acid (RA), found in several medicinal plants, is a natural phenol carboxylic acid with anti-inflammatory, antioxidants, analgesic, and anti-cancer activity [85,86,87]. Studies conducted by Hasanein et al. in 2014 showed that oral administration of RA has anti-hyperalgesic and antiallodynic action in experimental models of streptozotocin-induced diabetic rats [88]. Furthermore, *Agrimonia eupatoria* L., a medicinal plant used in traditional medicine, contains phenolic acids, flavonoids, triterpenes, and tannins, and is known for its antioxidant, antidiabetic, and antibacterial properties [89]. Lee et al., 2016 also showed the antinociceptive effect of *Agrimonia eupatoria* L. in the chemotherapy-neuropathic pain model, showing a lower paw withdrawal latency in the plantar test and pin-prick test, and a higher paw withdrawal threshold in the Randall–Selitto test compared to the neuropathic pain group [89]. 

Ginseng, the root of *Panax ginseng C.A*. Meyer, has been used extensively for the treatment of various ailments in traditional Asian medicine. Currently, ginsenoside monomers have been isolated, each with different effects [90]. Park et al. (2009) showed that ginseng extract and RB1, the most abating ginsenoid, appear to have anti-inflammatory effects [91]. In this regard, Lee et al., 2020 showed that saponin extract and Rb1 also have potential antinociceptive effects against neuropathic pain [90].

Medicinal plant extracts have antinociceptive and anti-inflammatory effects [92]; *Harpagophytum procumbens D.C*., a medicinal herb from southern Africa, has different therapeutic uses, including antioxidant properties [93]. Parenti et al. in 2015 observed, in rats with CCI, that the combined administration of *H. procumbens* and morphine could develop a synergistic effect against allodynia and hyperalgesia, evaluated by von Frey and plantar tests [94].

*Bacopa monnieri* (Linn.) Pennell possesses numerous properties, some of which are anti-dementia, cognitive enhancement, antidepressant, antiepileptic, anti-inflammatory, and neuroprotective. Specifically, *Bacopa monnieri* is known to attenuate opioid tolerance and increase opioid-induced analgesia. Furthermore, it was observed to have strong antinociceptive properties in relieving hyperalgesia and allodynia in the chronic constricting injury animal model of neuropathic pain. For these reasons, it could be considered a beneficial remedy for the management of painful neuropathic syndromes [95]. Gallic acid (GA), a natural polyphenolic found in green tea, grapes, and berries, showed antioxidant, anti-inflammatory, and anti-allergic properties associated with different pathways, including the inhibition of COX2 and the NF-kB signaling pathway [96,97]. Furthermore, GA showed antinociceptive effects during acute pain. Trevisan et al. in 2014 showed that oral administration of GA reduced mechanical allodynia and edema formation in an inflammatory model, and also decreased the nociception induced by a mechanical or cold stimulus in the CCI model of neuropathic pain [98].

Obesity is a significant factor causing insulin resistance and type 2 diabetes [99]. In particular, diabetic peripheral neuropathy is a disease of the vascular system, which can lead to ischemia and impaired nerve function. Studies observed that enalapril or α-lipoic acid monotherapies are effective in ameliorating diabetic peripheral neuropathy, vascular impairment of arterioles of the sciatic nerve [100], and oxidative stress [101,102]. Moreover, Holmes et al., in 2015, observed that dietary correction in obese and diabetic rats (12 weeks) with enalapril or menhaden oil improves neurological endpoints [103]. Indeed, diet treatment in obese rats with a high-fat diet containing enalapril or enriched with menhaden oil improved sensory nerve conduction velocity as well as intraepidermal nerve fiber density and sensitivity [103].

**Table 3:** Studies on natural products effects in several model of neuropathic pain.

References	Substances	Route of Administration	Gender Animals Strains	Pain Model	Behavioral Test	Results
**KOMATSU et al., 2018 [69]**	*Essential oil of Bergamot (BEO)* *(20 µg/paw)*	Subcutaneous injection (s.c) of BEO	Male Mice ddY	PSNL (partial sciatic nerve ligation)	Von Frey test	Attenuation of allodynia
**ILARI et al., 2020 [3] **	*Polyphenol fraction of Bergamot (BPF) (50 mg/Kg)*	Subcutaneous infusion of BPF by mini-pump	Male Rats Sprague–Dawley	Neuropathic pain induced by sciatic nerve injury (CCI)	-Von Frey test -Plantar test	Reduction in mechanical allodynia and thermal hyperalgesia
**ILARI et al., 2021 [71] **	*Polyphenol fraction of Bergamot (BPF) (25 mg/Kg)*	Intraperitoneal injection (i.p.) of BPF	Male Rats Sprague–Dawley	Neuropathic pain induced by administration of paclitaxel	-Von Frey test -Plantar test	Reduction in mechanical allodynia and thermal hyperalgesia
**IANNOTTA et al., 2021 [72] **	*N-Palmitoyl-D-glucosamine* *(PGA)* *(20 mg/Kg)*	Oral gavage	Male Mice CD1	Chemotrapy-induced peripheral neuropathy by oxaliplatin	-Von Frey -Cold plate	Prevention of mechanical allodynia and hyperalgesia
**TENCI et al., 2017 [73] **	*Lepidium meyenii Walp. (Maca) * *(10g/Kg)*	Oral gavage	Male Rats Sprague–Dawley	Neuropathic pain induced by administration of paclitaxel and oxaliplatin	-Cold plate	Reduction in thermal hyperalgesia
**BAGDAS et al., 2014 [74] **	*Chlorogenic Acid (5-caffeoylquinic acid, CGA) (100 mg/Kg)*	Intraperitoneal injection (i.p.)	Male Rats Wistar	Streptozotocin-induced diabetic neuropathic pain	-Randall–Selitto test	Prevention of mechanical allodynia
**LINHER-MELVILLE et al., 2020 [75] **	*Cannabil oil (CBD)* *(0.0833 mg/200gr rats) and Δ9-tetrahydrocannabinol (THC) (0.0167 mg/200 rats).*	Oral gavage	Male Rats Sprague–Dawley	Neuropathic pain by sciatic nerve cuff surgery	Von Frey test	Reduction in mechanical allodynia
**BELARDO et al., 2019 [77] **	*Cannabil oil (CBD)* *(30 μL of CBD dissolved in 10% hemp seed oil and natural tocopherols)*	Oral gavage	Male Mice C57BL/6	Traumatic brain injury (TBI)	Von Frey test	Reduction in mechanical allodynia
**GUIDA et al., 2015 [80] **	*Palmitoylethanolmide (PEA) (10 mg/Kg) and Oleoylethanolamide (OEA) (10 mg/Kg)*	Intraperitoneal injection (i.p.)	Male Mice CD1	Neuropathic pain induced by spared nerve injury (SNI)	-Plantar test -Dynamic plantar aesthesiometer	Reduction in mechanical allodynia and hyperalgesia
**RENNO et al., 2012 [81] **	*Epigallocatechin-3-Gallate* *(EGCG)* *(50 mg/Kg)*	Intraperitoneal injection (i.p.)	Male Rats Wistar	Neuropathic pain induced by sciatic nerve crush injury	-Von Frey test -Randall–Selitto test -Hotplate test	Reduction in mechanical allodynia and hyperalgesia
**XIFRò et al., 2015 [82] **	*Epigallocatechin-3-Gallate (EGCG) (50 mg/Kg)*	Intraperitoneal injection (i.p.) of EGCG	Female Mice Balb-c	Neuropathic pain induced by sciatic nerve injury (CCI)	Plantar test	Reduction in thermal hyperalgesia
**BOSCH-MOLA et al., 2017 [83] **	*Epigallocatechin-3-Gallate (EGCG) (50 mg/Kg)*	Intraperitoneal injection (i.p.) of EGCG	Female Mice Balb/c	Neuropathic pain induced by sciatic nerve injury (CCI)	Plantar test	Reduction in thermal hyperalgesia
**ISHOLA et al., 2014 [84] **	*Annona muricata Linn. (Soursop)* *(200 mg/Kg)*	Oral gavage	Male albino mice	Morphine induced pain	Hot plate test	Reduction in thermal hyperalgesia
**HASANEIN et al., 2014 [88] **	*Rosmarinic Acid (RA) (30 mg/Kg)*	Oral gavage (o.g.) of RA	Male Rats Wistar	Diabetic neuropathy induced by streptozotocin (STZ)	Tail flick latency	Reduction in hyperalgesia and allodynia
**LEE et al., 2016 [89] **	*Agrimonia eupatoria* L. *(200 mg/Kg)*	Oral gavage	Male Rats Sprague–Dawley	Cisplatin-induced neuropathic pain	-Pin-prick test -Randall–Selitto -Plantar test	Reduction in hyperalge
**LEE et al., 2021 [104] **	*Ginseng (Panax ginseng C.A. Meyer) (saponin extract 50 mg/Kg; Rb1 12.5 mg/Kg)*	Oral gavage	Male Rats Sprague–Dawley	Neuropathic pain induced by tail nerve injury (TNI) and spinal cord injury (SCI)	-Plantar test -Von Frey test	Reduction in allodynia and hyperalgesia
**PARENTI et al., 2015** **[94]** **SHAHID et al., 2017** **[95] **	*Harpagophytum procumbens* *(400–800 mg/Kg) plus morphine (3–5 mg/Kg)* *Bacopa monnieri (Linn.) Pennell* *(80 mg/Kg)*	Intraperitoneal injection (i.p.) Oral gavage	Male Rats ---- Male Rats Sprague–Dawley	Neuropathic pain induced by sciatic nerve injury (CCI) Neuropathic pain induced by sciatic nerve injury (CCI)	-Plantar test -Von Frey test -Von Frey test -Hot plate test	Reduction in allodynia and hyperalgesia Reduction in mechanical allodynia and hyperalgesia
**HOLMES et al., 2015 [103] **	*-Menhaden oil* *(replacing 50% of the high fat diet)*	Oral administration (containing in high fat diet)	Male Rats Sprague–Dawley	Diabetic neuropathy induced by streptozotocin (STZ)	Plantar test	Reduction in thermal hyperalgesia

### 3.3. Meta-Analysis

The systematic review identified 19 papers, divided into two separate meta-analyses according to behavioral analyses used for the identification of neuropathic pain (thermal hyperalgesia (sec) or mechanical (gr) hyperalgesia/allodynia). Articles which reported behavioral studies concerning both thermal and mechanical approaches were included in both meta-analyses. Strains and genders of mice/rats were considered.

#### 3.3.1. Meta-Analysis of Thermal Hyperalgesia

**Figure 4:**
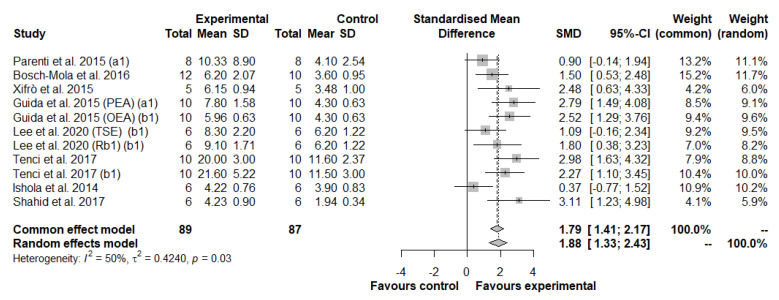
Forest plot showing the nutraceutical effects in thermal hyperalgesia by measuring the withdrawal latency in the injured paw. SD, standard deviation; SMD, standardized mean differences; CI, confidence interval.

**Figure 5:**
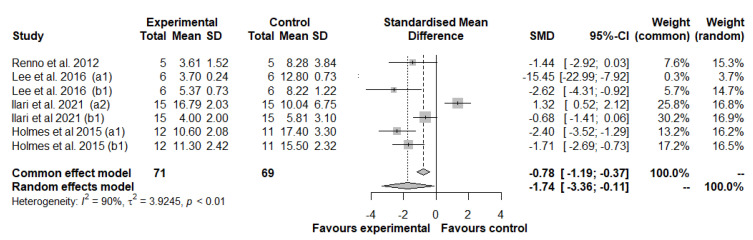
Forest plot showing the nutraceutical effects on thermal hyperalgesia by measuring the withdrawal latency in the two paws. SD, standard deviation; SMD, standardized mean differences; CI, confidence interval.

Based on pain assessment methods, we divided this meta-analysis into two further meta-analyses: the first measured the hyperalgesic threshold only on the injured paw (Figure 4), and the second measured the hyperalgesic threshold on both paws (Figure 5).

In the first meta-analysis, eleven studies, with a total of 89 animals in the experimental group and 87 animals in the control group, were included (Figure 4). Results generated by fixed model showed a strong beneficial effect of natural drugs on thermal hyperalgesia measured in the injured paw, compared to the control group (SMD: 1.79; 95% CI: 1.41 to 2.17; *p* < 0.0001). A low and statistically significant heterogeneity between studies was observed: I2 = 50%, *p* = 0.03 (Figure 4). Visual inspection of the funnel plot did not identify substantial asymmetry (Supplementary Figure S1), also confirmed by the Egger’s test (t = 2.05; *p* = 0.07).

Seven studies, with a total of 71 animals in the experimental group and 69 animals in the control group, were included in the second meta-analysis (Figure 5). Results generated by the random effects model showed a strong beneficial effect compared to the control group of natural drugs in thermal hyperalgesia measured in the two paws (SMD: −1.74; 95% CI: −3.36 to −0.11; *p* = 0.036). A high heterogeneity between studies was observed: I2 = 90%, *p* < 0.01. Visual inspection of the funnel plot identified substantial asymmetry, confirmed by the trim and fill method, with three added studies printed as open circles (*p* = 0.782) (Supplementary Figure S2), confirmed by Egger’s test (*t* = −2.37, *p* = 0.064).

##### Multivariate Meta-Regression Analysis in Thermal Hyperalgesia

Selected variables, such as publication year, animal species, behavioral test, pain model, and route of administration were tested as potential confounders in the meta-regression analysis. None of them resulted as statistically significant (data not shown).

#### 3.3.2. Meta-Analysis of Mechanical Allodynia/Hyperalgesia

**Figure 6:**
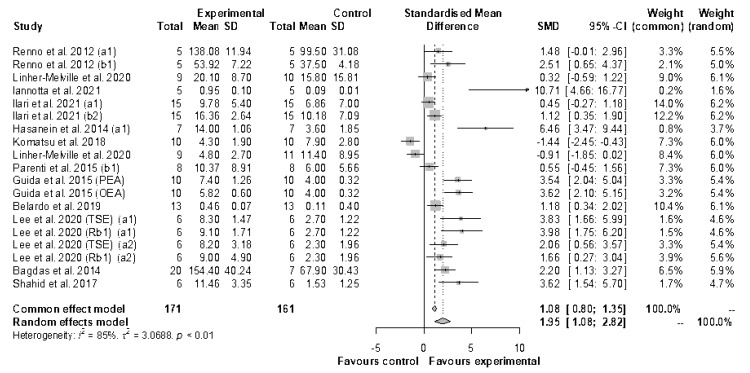
Forest plot showing the nutraceutical effects of reducing mechanical allodynia and hyperalgesia by measuring the withdrawal threshold in the injured paw. SD, standard deviation; SMD, standardized mean differences; CI, confidence interval.

A total of 19 studies were included in the meta-analysis of mechanical allodynia/hyperalgesia, including a total of 171 animals in experimental groups and 161 animals in the control groups (Figure 6). Results generated by the random-effects model showed a strong beneficial effect of natural drugs on mechanical allodynia/hyperalgesia measured in the injured paw, compared to untreated animals (SMD: 1.95, 95% CI: 1.08 to 2.82; *p* < 0.001). 

A high heterogeneity between studies (I2 = 85.0%; *p* < 0.01), was confirmed by the trim and fill method, with seven added studies (*p* = 0.172) (Supplementary Figure S3), and by the Egger’s test (t = 4.55; *p* = 0.0003). 

##### Subgroup Analysis and Univariate Meta-Regression in Mechanical Allodynia/Hyperalgesia

The high degree of heterogeneity observed in this meta-analysis allowed us to test different variables, such as publication year, animals, behavior, pain model, and route of administration, as potential confounders in the meta-regression analysis. In the univariate meta-regression, animals and their behavior were not significantly associated with the effect of natural drugs on pain.

**Figure 7:**
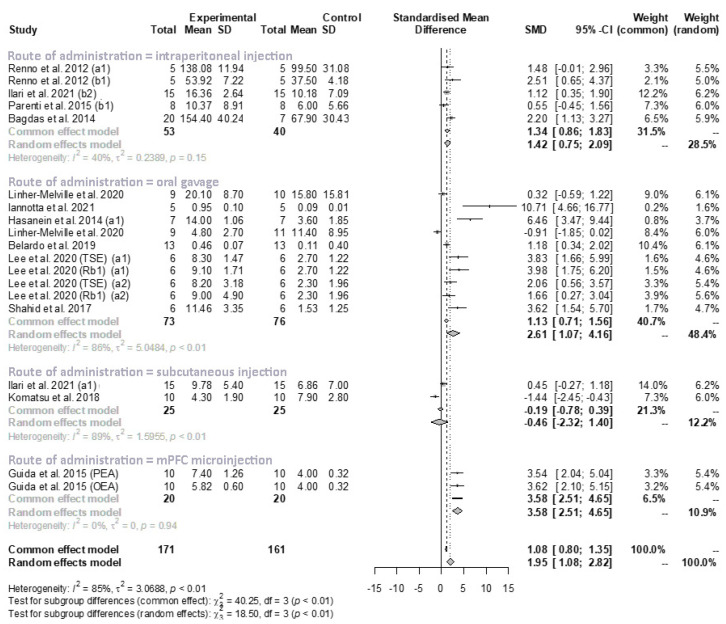
Forest plot showing the nutraceutical effects in the subgroup (route of administration) of mechanical allodynia/hyperalgesia by measuring the withdrawal threshold in the injured paw. SD, standard deviation; SMD, standardized mean differences; CI, confidence interval.

In order to further investigate the residual heterogeneity observed in the meta-analysis of mechanical allodynia/hyperalgesia, route of administration (Figure 7) and pain model (Figure 7) were divided into small subgroups and re-analyzed. With regard to the route of administration, mPFC microinjection had a strong effect (SMD: 3.58; 95% CI: 2.51 to 4.65) and oral gavage (SMD: 2.61; 95% CI: 1.07 to 4.16) had a moderate effect, while intraperitoneal injection (SMD: 1.42; 95% CI: 0.75 to 2.09) had a lower and insignificant effect, and subcutaneous administration was not associated with any effect (Figure 7). Heterogeneity was generally low or very low, apart from with oral gavage (I2 = 86%, *p* < 0.01). 

**Figure 8:**
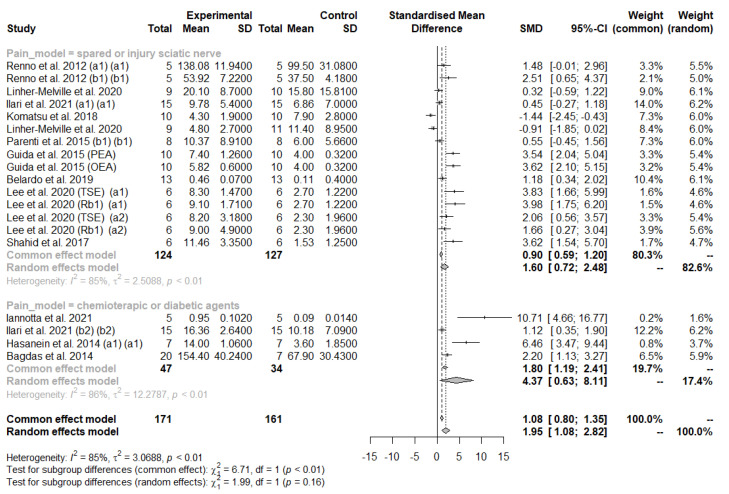
Forest plot showing the nutraceutical effects in the subgroup (pain model) of mechanical allodynia/hyperalgesia, observed by measuring the withdrawal threshold in the injured paw. SD, standard deviation; SMD, standardized mean differences; CI, confidence.

In the meta-analysis of the pain model subgroup, we observed that neuropathic pain induced by chemotherapy or diabetic agents had a stronger effect (SMD: 4.7, 95% CI: 0.63 to 8.11) than that induced by injury of the sciatic nerve (SMD: 1.60, 95% CI: 0.72 to 2.48) (Figure 8). A high heterogeneity between studies was observed (I2 = 86.0%, *p* < 0.01 and I2 = 85.0%, *p* < 0.01, respectively) (Figure 8).

Meta-regression analyses investigated whether route of administration and pain model were positively or negatively associated with intervention effects.

Specifically, the univariate meta-regression for route of administration did not show a statistically significant difference between subgroups (QM = 7.11, *p* = 0.07). Likewise, the univariate meta-regression for pain model also did not show a statistically significant difference between subgroups (QM = 2.52, *p* = 0.11).

## 4. Discussion

The present meta-analysis provides a comprehensive report on the effect of natural drugs in controlling neuropathic pain. Through meta-regression, we evaluated the confounding factors (animal model of pain, type of animal, route of administration, and different pain measurements) which could possibly be responsible for the heterogeneity observed in the results. 

Neuropathic pain is a health problem affecting many people around the world, with critical socioeconomic effects. Regarding the mechanisms, the role of oxidative stress and the inflammatory response has been extensively described [105]. Furthermore, pharmacological and non-pharmacological treatments are available for chronic pain, although the limited efficacy and side effects these therapies make their use controversial [8]. Therefore, it becomes urgent to discover new, safe, and effective strategies to prevent this condition.

In recent years, new therapeutic options to contrast neuropathic pain have been investigated; among them are natural products, especially medicinal herbs [55]. Phytochemicals prevent diseases due to their antibacterial, antifungal, anti-inflammatory, diuretic, and anesthetic effects. Additionally, some phytochemicals protect against oxidative stress damage, and thus inhibit different types of pain. In this regard, numerous studies are currently focusing on the characterization and application of natural agents in various diseases for the reduction in and/or elimination of free radicals [17].

This systematic review examined the effect of nutraceuticals, mainly antioxidants, on the reduction in neuropathic pain measured through thermal hyperalgesia or mechanical allodynia/hyperalgesia. 

In total, 19 preclinical studies met the inclusion criteria. Fifteen natural compounds groups were included: bergamot (*Citrus bergamia*) (*n* = 3), cannabinoid oil (*n* = 2), N-Palmitoyl-D-glucosamine (*n* = 1), palmitoylethanolmide and oleoylethanolamide (*n* = 1), epigallocatechin-3-Gallate (*n* = 3), rosmarinic acid (*n* = 1), *Agrimonia eupatoria* L. (*n* = 1), *Harpagophytum procumbens* (*n* = 1), *Lepidium meyenii Walp.* (Maca) (*n* = 1), chlorogenic acid (5-caffeoylquinic acid, CGA) (*n* = 1), *Annona muricata Linn*. (Soursop) (*n* = 1), ginseng (*Panax ginseng C.A. Meyer*) (*n* = 1), *Bacopa monnieri* (Linn.) pennell (*n* = 1), and menhaden oil (*n* = 1). All studies compared the effects of natural treatment on neuropathic pain in mice (*n* = 7) or rats (*n* = 12), which were mostly male. 

Various mechanisms are involved in the onset of neuropathic pain, the most important of which are mechanically induced pain neuropathy, diabetes, and chemotherapy [3,71]. Therefore, the present study includes data related to these mechanisms, and through the analysis of thermal hyperalgesia and mechanical allodynia/hyperalgesia, nutraceuticals seem to improve the symptoms of neuropathic pain caused by all of these mechanisms.

In this setting, it the effect of the natural drugs was compared in the following three neuropathic pain models in animals: CCI, SNI, PSNL, sciatic nerve crush and sciatic nerve cuff surgery (peripheral neuropathic pain), brain trauma (central neuropathic pain), streptozotocin-induced neuropathic pain (diabetic induced neuropathic pain), and paclitaxel-oxaliplatin- and cisplatin-induced neuropathic pain (chemotherapy induced neuropathic pain).

Meta-analyses showed the beneficial role of nutraceuticals in animals with neuropathic pain by evaluating the effect of treatment on the thermal hyperalgesia measured in the injured paw (SMD: 1.79; 95% CI: 1.41 to 2.17; *p* < 0.0001) and in the two paws (SMD: −1.74; 95% CI: −3.36 to −0.11; *p* = 0.036), or by targeting mechanical allodynia and hyperalgesia (SMD: 1.95; 95% CI: 1.08 to 2.82; *p* < 0.001). 

However, the substantial heterogeneity between studies allowed us to perform subgroup analyses to discover the source of this heterogeneity.

These analyses revealed that the administration of natural drugs improved thermal hyperalgesia regardless of the type of animal, the behavioral test, or the route of administration. Furthermore, the analysis showed that natural drug administration improved mechanical allodynia/hyperalgesia symptoms primarily in chemotherapy or diabetes-induced neuropathic pain (although only four studies used these mechanisms), compared to mechanically induced neuropathic pain (fifteen studies). More studies are needed to define the best pain model for the effectiveness of natural drugs.

The route of administration of the natural compound was also evaluated in this study. In particular, the method of drug administration in the analysis of mechanical hyperalgesia/allodynia was subcutaneous in two studies, intraperitoneal in five studies, and by gavage in ten studies, while only one author used m-PFC microinjection. The analysis revealed that m-PFC microinjection and oral gavage could be the main routes for natural drugs in neuropathic pain. However, given the few studies reviewed, these results require further investigation.

However, despite the heterogeneity found, all of the results of the experimental studies on the use of nutraceutical compounds provide substantial mechanistic support to the use of these therapeutic options for managing persistent pain in clinical and rehabilitative settings.

### Limits and Strengths of the Study

Possible methodological limitations of this systematic review and meta-analysis need to be considered: some data were collected from the same study, which could influence the results with regard to the efficacy of the natural compounds in neuropathic pain. All studies included in this review showed different experimental designs. The natural compounds identified derive from several families, including capnellene, epigallocatechin-3-gallate, *Agrimonia eupatoria L*., essential oil of bergamot, gallic acid, rosmarinic acid, menaden oil, cannabiloil oil, N-Palmitoyl-D-glucosamine, polyphenolic fraction of bergamot, *Harpagophytum procumbens*, *Lepidium meyenii Walp*., CGA, *Annona muricata Linn*. (Soursop), ginseng, *Bacopa monnieri* (Linn.) pennell, Palmitoylethanolamide (PEA) and oleoylethanolamide (OEA), thus increasing heterogeneity. Another limitation of the present work was the low number of studies on the efficacy of natural drugs in managing thermal hyperalgesia and mechanical allodynia/hyperalgesia, due to a lack of data from other works.

## 5. Conclusions

The results of this systematic review and meta-analysis clearly illustrate the efficacy of nutraceutical compounds in preclinical neuropathic pain models. In particular, in vivo pain models could represent an important starting point for increasing the translational impact on pain research. Of course, new research is needed to improve the homogeneity of the studies. It is important to note that although there was much heterogeneity, all of the results found a beneficial effect of drugs in neuropathic pain induced in different ways, thus showing the importance of the use of the nutraceuticals and the validity of this study model.

## Figures and Tables

**Table 1:** Pharmacological treatment in neuropathic pain.

Drugs	Mechanism of Action	Side Effect	Reference
**Triciclic antidepressant (TCA)**	- Blockade sodium channel - Anticholinergic effects	somnolence, dizziness, suicide risk, urinary retention	[9]
**Serotonin-noradrenalin reuptake inhibitors**	- Serotonin and noradrenaline reuptake - Inhibition potential calcium	nausea, hypertension, ataxia, lethargy	[14]
**Anticonvulsant**	- Channel-blocking effects - Decrease central sensitization	sedation, dizziness, oedema	[11]
**Local anesthetics** **- Licodaine**	- Sodium channel blockade	local erythema, rash	[13]
**Opioids**	- µ-receptor agonist	nausea, vomiting, somnolence, dizziness	[13]

**Table 2:** Polyphenols classification, food origin, and potential health benefits.

**Polyphenols**.	***Flavonoids*** - **Food origin:** Fruits, Vegetable, Plants - **Potential health benefit**: Asthma, Cancer, Artery diseases	**Flavones**
		**Flavanols**
		**Isoflavones**
		**Flavanones**
		**Anthocyanidis**
		**Flavanols**
	***Phenolic Acid*** - ***Food origin:*** Plants, Vegetables - ***Potential health benefit:*** Cancer, Inflammation	**Hydroxycimamic Acids**
		**Hydrobenzoic Acids**
	***Lignans*** - ***Food origin:*** Plants, Vegetables - ***Potential health benefit:*** Heart diseases, Osteoporosis, Breast cancer, Menopausal symptoms	**Secosolariciresinal diglucoside**
	***Stilbenes*** - ***Food origin:*** Fruits - ***Potential health benefit:*** Cancer, Ischemic damage	**Resveratrol**

## Data Availability

Data is contained within the article and Supplementary Materials.

## Supplementary Materials

The following supporting information can be downloaded at: https://www.mdpi.com/article/10.3390/antiox11122361/s1, Figure S1: Funnel plot for meta-analysis on the effects of natural drugs in neuropathic pain; Figure S2: Funnel plot for meta-analysis on the effects of natural drugs in neuropathic pain after applying the trim-and fill method. The filled -in-study results are printed as open circles.; Figure S3: Funnel plot for meta-analysis on the effects of natural in neuropathic pain after applying the trim-and fill method. The filled -in-study results are printed as open circles.
