# Supplementary material for: A Systematic Review and Meta-Analysis on the Role of Nutraceuticals in the Management of Neuropathic Pain in In Vivo Studies
Source: Antioxidants (Basel). 2022 Nov 28;11(12):2361. doi: 10.3390/antiox11122361 (PMC9774415; doi:10.3390/antiox11122361)
Supplement: Supplementary file 1 [file antioxidants-11-02361-s001.zip › antioxidants-1975881-supplementary.pdf]

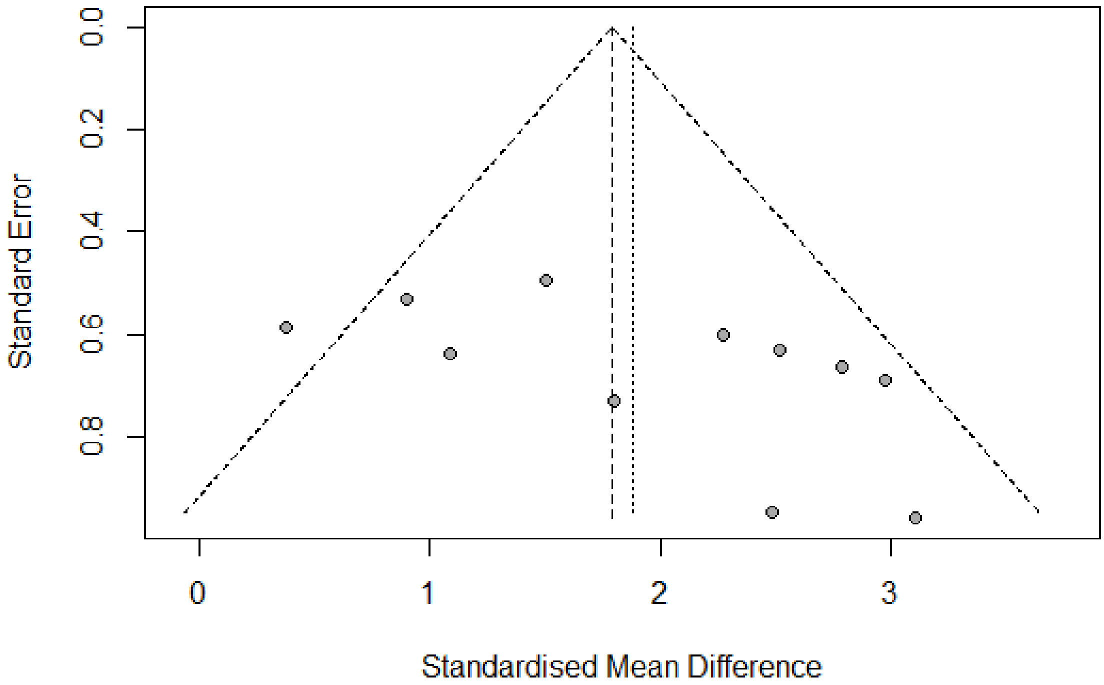

Supplementary Figure S1. Funnel plot for meta-analysis on the effects of natural drugs in neuropathic pain.

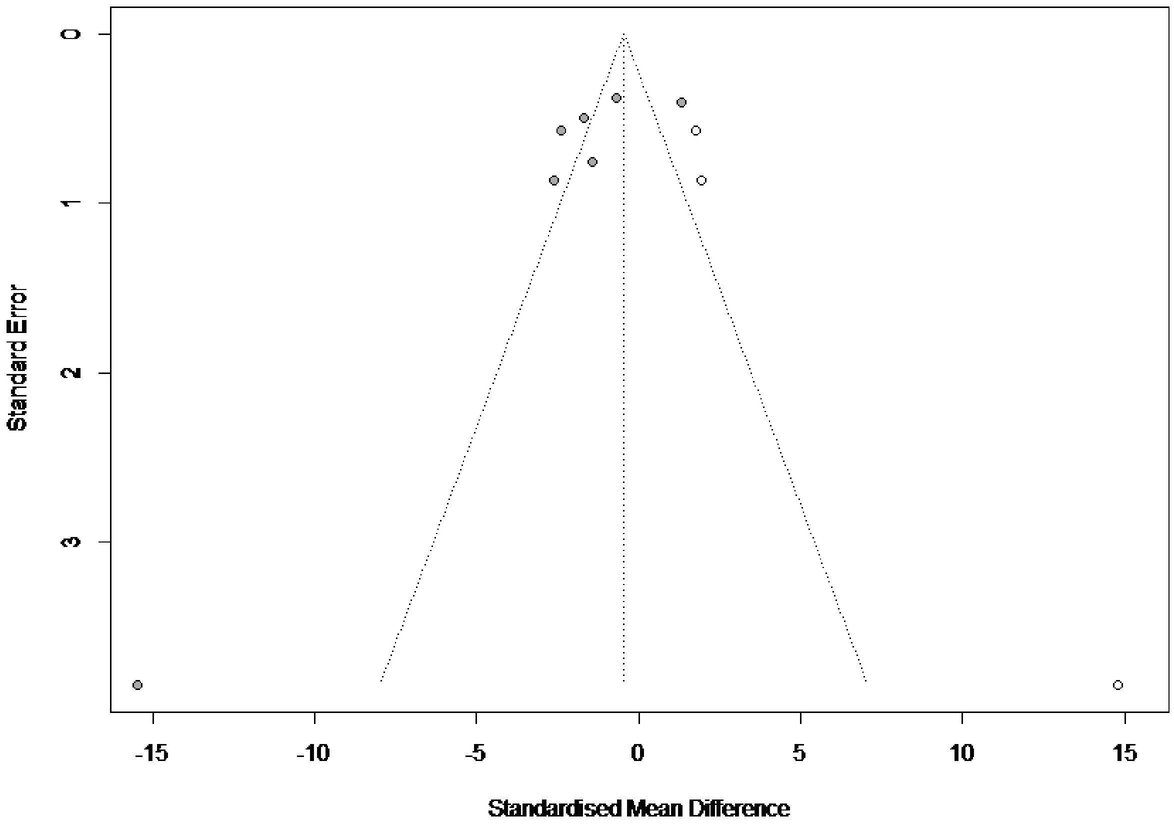

Supplementary Figure S2. Funnel plot for meta-analysis on the effects of natural drugs in neuropathic pain after applying the trim-and fill method. The filled -in-study results are printed as open circles.

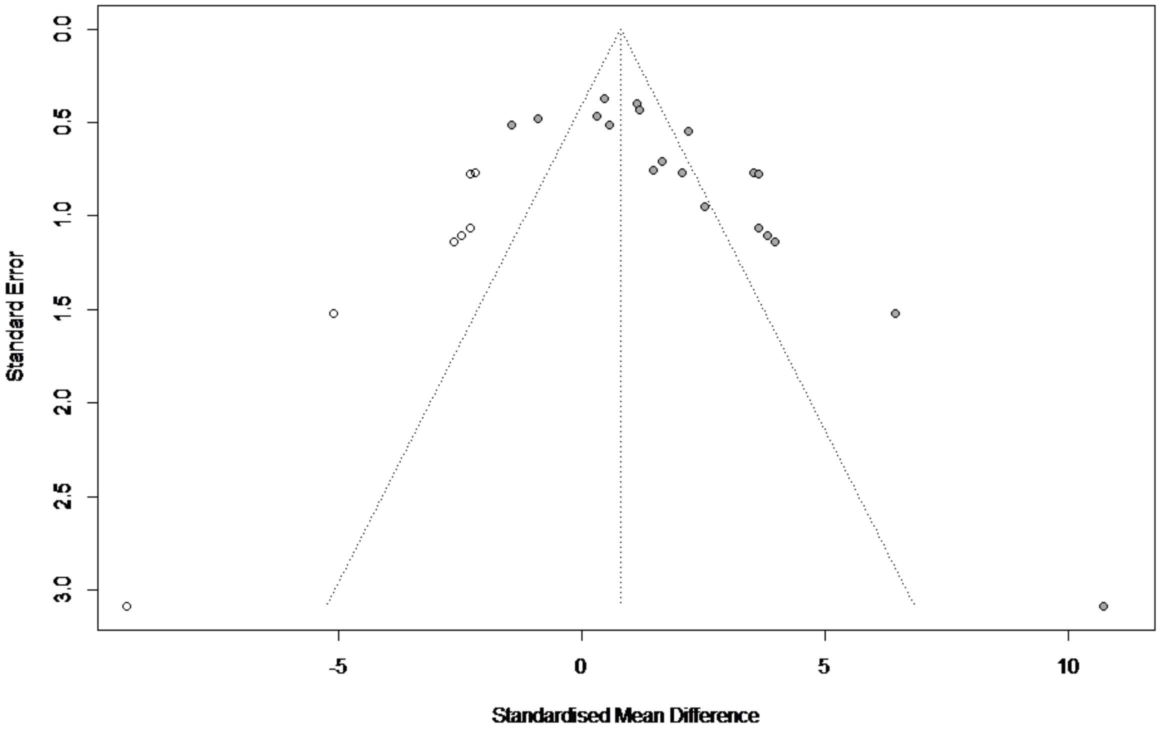

Supplementary Figure S3. Funnel plot for meta-analysis on the effects of natural in neuropathic pain after applying the trim-and fill method. The filled -in-study results are printed as open circles.
